# Cafestol overcomes ABT-737 resistance in Mcl-1-overexpressed renal carcinoma Caki cells through downregulation of Mcl-1 expression and upregulation of Bim expression

**DOI:** 10.1038/cddis.2014.472

**Published:** 2014-11-06

**Authors:** S M Woo, K-j Min, B R Seo, J-O Nam, K S Choi, Y H Yoo, T K Kwon

**Affiliations:** 1 Department of Immunology, School of Medicine, Keimyung University, 2800 Dalgubeoldaero, Dalseo-Gu, Daegu 704-701, South Korea; 2 Department of Ecological Environment Conservation, Kyungpook National University, Sangju-si, Gyeongsangbuk-do 742-711, South Korea; 3 Department of Biochemistry, Ajou University School of Medicine, 5 Woncheon-Dong, Paldal-Gu, Suwon 442-749, South Korea; 4 Department of Anatomy and Cell Biology and Mitochondria Hub Regulation Center, Dong-A University College of Medicine, Busan 602-714, South Korea

## Abstract

Although ABT-737, a small-molecule Bcl-2/Bcl-xL inhibitor, has recently emerged as a novel cancer therapeutic agent, ABT-737-induced apoptosis is often blocked in several types of cancer cells with elevated expression of Mcl-1. Cafestol, one of the major compounds in coffee beans, has been reported to have anti-carcinogenic activity and tumor cell growth-inhibitory activity, and we examined whether cafestol could overcome resistance against ABT-737 in Mcl-1-overexpressed human renal carcinoma Caki cells. ABT-737 alone had no effect on apoptosis, but cafestol markedly enhanced ABT-737-mediated apoptosis in Mcl-1-overexpressed Caki cells, human glioma U251MG cells, and human breast carcinoma MDA-MB231 cells. By contrast, co-treatment with ABT-737 and cafestol did not induce apoptosis in normal human skin fibroblast. Furthermore, combined treatment with cafestol and ABT-737 markedly reduced tumor growth compared with either drug alone in xenograft models. We found that cafestol inhibited Mcl-1 protein expression, which is important for ABT-737 resistance, through promotion of protein degradation. Moreover, cafestol increased Bim expression, and siRNA-mediated suppression of Bim expression reduced the apoptosis induced by cafestol plus ABT-737. Taken together, cafestol may be effectively used to enhance ABT-737 sensitivity in cancer therapy via downregulation of Mcl-1 expression and upregulation of Bim expression.

Bcl-2 family is classified as two groups, which is characterized by the presence of Bcl-2 homology (BH) domains: (1) anti-apoptotic proteins, containing the BH1–4 domains (Bcl-2, Bcl-X_L_, Bcl-w, and Mcl-1), and (2) pro-apoptotic proteins, containing the BH1–3 domains (Bax, Bak, and Bok), and BH3-only proteins, containing only BH3 domain (B-cell lymphoma 2 interacting mediator of cell death (Bim), p53 upregulated modulator of apoptosis (PUMA), and NOXA).^1, 2^ The balance of expression of the Bcl-2 family is important for the modulation of apoptosis in cancer cells. Bax and/or Bak are activated by apoptotic stimuli and then oligomerized on mitochondria. After oligomerization, cytochrome *c* is released from mitochondria to cytosol. Cytosolic cytochrome *c* binds with apoptotic protease activating factor-1 (Apaf-1) and then activates caspase signaling.^3, 4^ Anti-apoptotic Bcl-2 proteins sequester pro-apoptotic Bcl-2 proteins, thus maintaining mitochondrial integrity.^3, 4^ BH3-only protein antagonizes anti-apoptotic Bcl-2 proteins or activates pro-apoptotic proteins, resulting in induction of apoptosis.^5^

ABT-737 has been developed as a small-molecule BH3 mimetic. ABT-737 directly binds to Bcl-2, Bcl-xL, and Bcl-w with very high affinity, and antagonizes sequestration of Bax and Bak, which results in induction of apoptosis in cancer cells.^6, 7^ However, ABT-737 has very low affinity for Mcl-1, so cancer cells with high levels of Mcl-1 are resistant to ABT-737.^8, 9^ To increase sensitivity to ABT-737, a large number of studies have developed strategies using multiple drugs, which modulate the expression and activity of Mcl-1.^10, 11, 12, 13, 14, 15^ Studies have shown that combined treatment with ABT-737 and other drugs, which have the ability to reduce Mcl-1 expression, is important for improvement of the anti-cancer effect by ABT-737.

Cafestol, as a diterpene molecule found in the beans of *Coffea Arabica*, has a wide variety of activities, including anti-inflammatory,^16, 17^ anti-angiogenic,^18^ anti-carcinogenic,^19^ and anti-tumor properties.^20^ In malignant pleural mesothelioma, cafestol has been shown to inhibit the transcriptional activity and protein expression of Mcl-1, survivin, and cyclin D1 and then induce apoptosis.^21^ Furthermore, we recently showed that cafestol increased apoptosis in several types of cancer cells through through downregulation of anti-apoptotic proteins (Bcl-2 and Mcl-1) and inhibition of Akt phosphorylation.^22^ As downregulation of Mcl-1 expression has been known to increase the sensitivity to ABT-737 in multiple cancer cells,^10, 12, 23^ treatment with cafestol may be a promising agent, which can overcome resistance of cancer cells to ABT-737.

In this study, we aimed to investigate whether induction of cafestol plus ABT-737 mediated apoptosis and to identify molecular mechanisms of cafestol to overcome resistance against ABT-737 in Mcl-1-overexpressed Caki cells.

## Results

### Effect of ABT-737-mediated apoptosis in Mcl-1-overexpressed Caki cells

ABT-737 directly binds to anti-apoptotic Bcl-2 protein (Bcl-2, Bcl-xL, and Bcl-w) and induces apoptosis,^6, 7^ but cancer cells with high expression levels of Mcl-1 have been reported to be resistant to ABT-737.^8, 9^ In an attempt to develop the therapeutic strategy to overcome ABT-737 resistance, we first tested whether ABT-737 differently regulate apoptosis in Caki/vector, Bcl-2-overexpressed Caki (Caki/Bcl-2), and Mcl-1-overexpressed Caki cells (Caki/Mcl-1). ABT-737 dose-dependently induced morphological features of apoptosis in Caki/vector and Caki/Bcl-2 cells (Supplementary Figure S1A). However, ABT-737 did not alter the morphologies of Caki/Mcl-1 cells (Supplementary Figure S1A). Furthermore, while ABT-737 increased sub-G1 population and the cleaved form of PARP in Caki/vector and Caki/Bcl-2 cells, ABT-737-mediated cell death rarely occurred in Caki/Mcl-1 cells (Supplementary Figures S1B and C). These results supported previous studies that Mcl-1 inhibits pro-apoptotic function of ABT-737.

### Cafestol synergizes ABT-737-induced apoptosis

**Figure 1:**
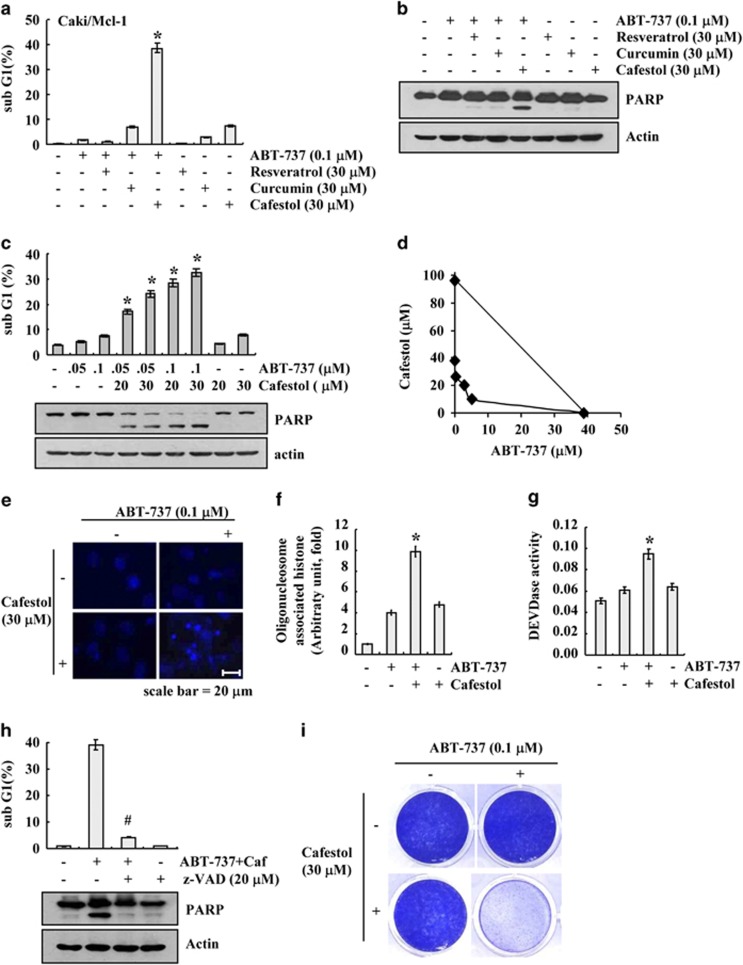
Cafestol sensitizes ABT-737-induced apoptosis in Mcl-1-overexpressed Caki cells. (**a** and **b**) Mcl-1-overexpressed cells (Caki/Mcl-1) were treated with 30 *μ*M resveratrol, 30 *μ*M curcumin, and 30 *μ*M cafestol in the presence or absence of 0.1 *μ*M ABT-737 for 24 h. (**c**) Mcl-1-overexpressed cells (Caki/Mcl-1) were treated with the indicated concentrations of cafestol and/or ABT-737 for 24 h. (**d**) Isoboles were obtained by plotting the combined concentrations of each drug required to produce 50% cell death. The straight line connecting the IC_50_ values obtained for two agents when applied alone corresponds to an additivity of their independent effects. Values below this line indicate synergy, whereas values above this line indicate antagonism. (**e**–**g**) Mcl-1-overexpressed cells (Caki/Mcl-1) were treated with 30 *μ*M cafestol and 0.1 *μ*M ABT-737 for 24 h. The condensation and fragmentation of the nuclei were detected by staining with DAPI (**e**). DNA fragmentation was determined using a DNA fragmentation detection kit (**f**). DEVDase (caspase-3) activity was determined using a caspase assay kit (**g**). (**h**) Mcl-1-overexpressed cells (Caki/Mcl-1) were pretreated with 20 *μ*M z-VAD-fmk for 30 min, then cafestol (Caf) and ABT-737 were added, and allowed to react for 24 h. (**i**) Effect of combined treatment with cafestol and ABT-737 on long-term survival. Mcl-1-overexpressed cells (Caki/Mcl-1) were treated with 30 *μ*M cafestol in the presence or absence of 0.1 *μ*M ABT-737 for 3 days. Clonogenic survival was determined by staining colonies with crystal violet and visualized with 0.4% coomassie blue by a digital camera. The level of apoptosis was analyzed by measuring the sub-G1 fraction by flow cytometry (**a**, **c**, and **h**, upper panel). Equal amounts of cell lysates (60 *μ*g) were separated by gel electrophoresis and analyzed by western blotting for poly ADP-ribose polymerase (PARP) cleavage (**b**, **c**, and **h**, lower panel). Actin served as a control for protein loading. The values in panels (**a**, **c**, **f**, **g**, and **h**) represent the mean±S.D. from three independent samples. *P<0.05 compared with ABT-737 alone and cafestol alone. ^#^*P*<0.01 compared with ABT-737 plus cafestol. The data represent three independent experiments

We next examined whether the resistance of cancer cells overexpressing Mcl-1 to ABT-737 could be overcome by co-treatment with well-known chemopreventive agents, including resveratrol, curcumin, or cafestol. As shown in Figures 1a and b, when Caki/Mcl-1 cells were treated with ABT-737 or chemopreventive agent alone, cell death rarely occurred. However, combined treatment with cafestol and ABT-737 resulted in induction of sub-G1 population and PARP cleavage in a dose-dependent manner (Figures 1a–c). Next, we examined whether combined treatment with ABT-737 and cafestol have synergistic effects. The isobologram analysis suggested that combined treatment with ABT-737 and cafestol have synergistic effects (Figure 1d). In addition, co-treatment with ABT-737 and cafestol increased chromatin damage in the nuclei and cytoplasmic histone-associated DNA fragmentation, indicating the key characteristic of apoptosis (Figures 1e and f). Next, we investigated whether the combined treatment with ABT-737 and cafestol gave rise to the activation of caspase-3. Exposure of Caki/Mcl-1 cells to cafestol plus ABT-737 increased caspase-3 activity (Figure 1g). To confirm whether the cell death induced by ABT-737 plus cafestol is dependent on caspase-3, we examined the effect of pretreatment with z-VAD-fmk, a pan-caspase inhibitor. As shown in Figure 1h, z-VAD completely inhibited the apoptosis and also blocked the PARP cleavage. Furthermore, we examined the effect of ABT-737 plus cafestol on long-term survival using clonogenic assay. Treatment with ABT-737 alone and cafestol alone did not affect colony formation. In contrast, combined treatment with ABT-737 plus cafestol markedly inhibited colony formation (Figure 1i). Therefore, these data indicated that cafestol increases the sensitivity to ABT-737-mediated apoptosis in Caki/Mcl-1 cells.

### Effect of combined treatment with cafestol and ABT-737 on other cancer cells and normal cells

**Figure 2:**
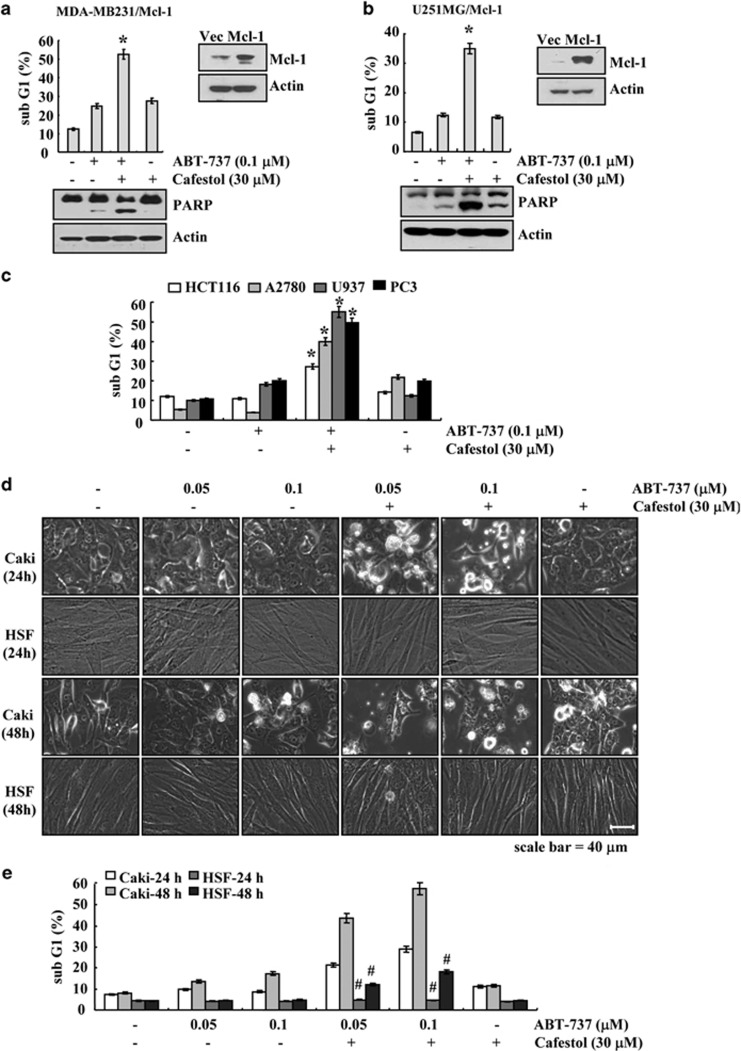
Effects of cafestol on ABT-737-induced apoptosis in other cell lines. (**a** and **b**) MDA-MB231 and U251MG cells were transiently transfected with pFLAG-CMV4 vector or pFLAG-CMV4/Mcl-1. Twenty-four hours after transfection, cells were treated with 0.1 *μ*M ABT-737 in the presence or absence of 30 *μ*M cafestol for 18 h. (**c**) HCT116, U937, PC3, and A2780 cells were treated with 0.1 *μ*M ABT-737 in the presence or absence of 30 *μ*M cafestol for 24 h. (**d** and **e**) Caki and HSF cells were treated with the indicated concentrations of ABT-737 in the presence or absence of 30 *μ*M cafestol for 24 h (upper panel) and 48 h (lower panel). The level of apoptosis was analyzed by measuring the sub-G1 fraction by flow cytometry (upper panel of **a** and **b**, **c**, and **e**). Equal amounts of cell lysates (60 *μ*g) were separated by gel electrophoresis and analyzed by western blotting for poly ADP-ribose polymerase (PARP) (**a** and **b**). Actin served as a control for protein loadings. The values in panels (**a**, **b**, **c**, and **e**) represent the mean±S.D. from three independent samples. *P<0.05 compared with ABT-737 alone and cafestol alone. ^#^*P*<0.01 compared with Caki. The data represent three independent experiments

To further investigate whether combined treatment with cafestol and ABT-737 induces apoptosis in other cancer cell types, human breast carcinoma cells (MDA-MB231), and human glioma cells (U251MG), cells were transiently transfected with pFLAG-CMV4/Mcl-1. When MDA-MB231/Mcl-1 and U251MG/Mcl-1 cells were treated with cafestol and ABT-737, sub-G1 population and the cleaved form of PARP were markedly increased, compared with the treatment with cafestol or ABT-737 alone (Figures 2a and b). Furthermore, to investigate the anti-cancer effect of ABT-737 and cafestol on other carcinoma cells, we used other cancer cells (human colon carcinoma cells (HCT116), human leukemia cells (U937), and human prostate carcinoma (PC3) cells, and human ovarian carcinoma cells (A2780). As shown in Figure 2c, combined treatment with ABT-737 and cafestol induced apoptosis in HCT116, U937, PC3, and A2780 cells. On the other hand, combined treatment with cafestol and ABT-737 did not affect the morphology or viability of human skin fibroblasts (HSF) cells (Figures 2d and e). These data suggest that cafestol increases sensitivity to ABT-737-mediated apoptosis also in other cancer cells but not in normal cells.

### Combined treatment with cafestol and ABT-737 inhibits tumor growth *in vivo*

**Figure 3:**
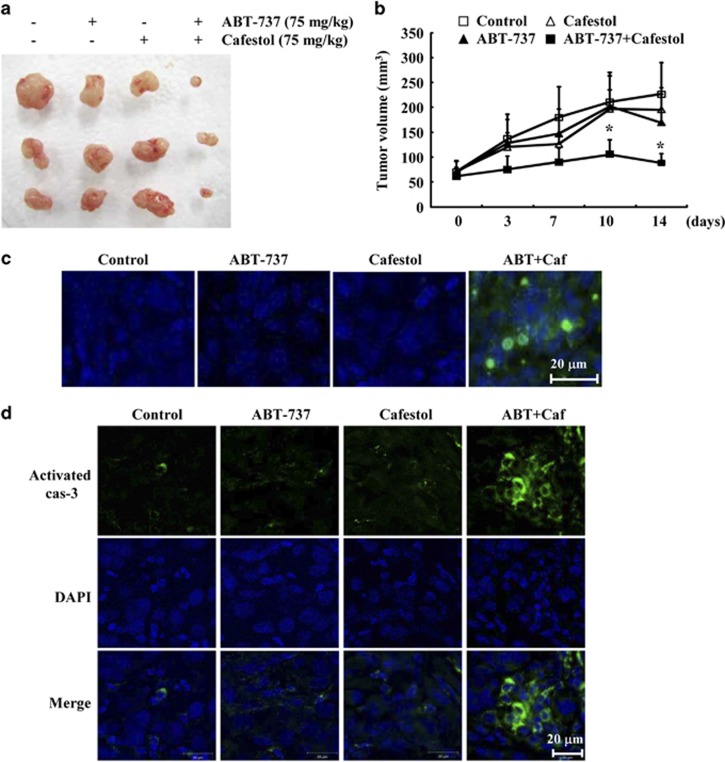
Tumor growth *in vivo* is reduced by combined treatment with cafestol and ABT-737. Nude mice were s.c. inoculated with Mcl-1-overexpressed cells. Tumor volume was monitored during the following treatments: vehicle, ABT-737 (75 mg/kg; i.p.), cafestol (75 mg/ml, i.p.), or ABT-737 plus cafestol for 14 days. (**a**) Tumor size shows the size of the dissected out tumors. (**b**) Graph shows tumor volume changes. Number of animals per group=7. Data are means±S.E. (*n*=7). (**c**) Representative images of tumor sections that were analyzed by TUNEL assay. Nuclear staining was performed with DAPI. (**d**) Immunohistochemical analysis of activated caspase-3

Next, we investigated the anti-cancer effect of combined treatment with cafestol and ABT-737 *in vivo* xenograft model. Caki/Mcl-1 cells injected subcutaneously (s.c.) into the right flank were established. Mice bearing tumor were treated with cafestol alone, ABT-737 alone, or as a combined treatment with cafestol and ABT-737. As shown in Figures 3a and b, combined treatment markedly suppressed tumor growth compared with vehicle, ABT-737 alone, and cafestol alone. Terminal deoxynucleotide transferase (TdT)-mediated dUTP nick-end labeling (TUNEL) analysis showed that combined treatment with cafestol and ABT-737 increased cell death (Figure 3c). Moreover, immunohistochemical staining of tumor tissues showed that combined treatment increased activated caspase-3 (Figure 3d). These results suggest that combined treatment with cafestol and ABT-737 inhibits tumor growth and induces apoptosis *in vivo*.

### Downregulation of Mcl-1 expression is associated with cafestol-induced ABT-737-mediated apoptosis

**Figure 4:**
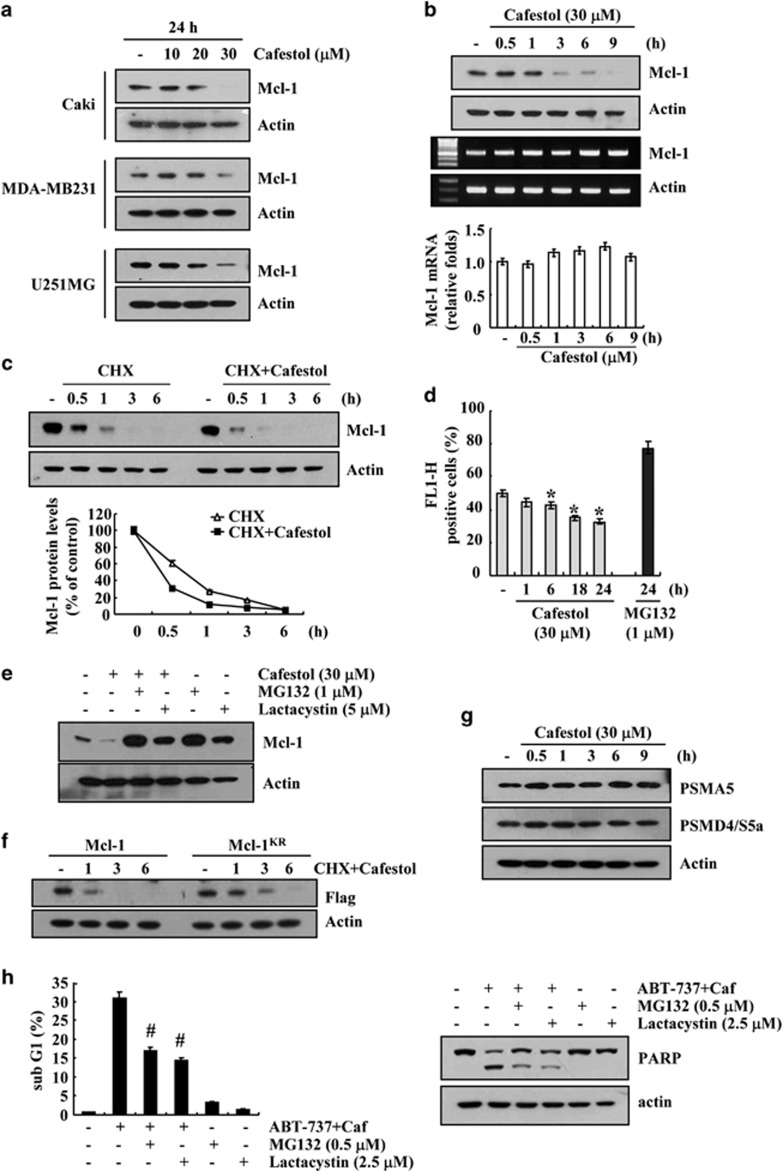
Cafestol modulates Mcl-1 expression at the posttranslational level. (**a**) Caki, MDA-MB231, and U251MG cells were treated with the indicated concentrations of cafestol for 24 h. (**b**) Caki cells were treated with 30 *μ*M cafestol for the indicated time periods. The levels of expression of Mcl-1 protein and mRNA were determined by western blotting, reverse transcriptase–PCR (RT-PCR), and quantitative PCR (qPCR), respectively. (**c**) Caki cells were pretreated with 20 *μ*g/ml CHX for 30 min and then treated with 30 *μ*M cafestol for the indicated time periods. The intensity of Mcl-1 protein bands was measured using the JAVA image-processing program ImageJ (http://rsb.info.nih.gov/ij) (lower panel). (**d**) Caki cells were transfected with the proteasome sensor vector (ZsProSensor-1) and treated with 30 *μ*M cafestol or 1 *μ*M MG132 for the indicated time periods. Proteasome activity was analyzed with FACS analysis. (**e**) Caki cells were pretreated with 1 *μ*M MG132 or 5 *μ*M lactacystin for 30 min and then treated with 30 *μ*M cafestol for 24 h. (**f**) Caki cells were transiently transfected with Flag-Mcl-1 and Flag-Mcl-1^KR^. Twenty-four hours after transfection, cells were treated with 20 *μ*g/ml CHX and 30 *μ*M cafestol for the indicated time periods. (**g**) Caki cells were treated with 30 *μ*M cafestol for the indicated time periods. (**h**) Caki cells were pretreated with 1 *μ*M MG132 or 5 *μ*M lactacystin for 30 min and then treated with 30 *μ*M cafestol and 0.1 *μ*M ABT-737 for 24 h. The level of apoptosis was analyzed by measuring the sub-G1 fraction by flow cytometry. The levels of expression of Mcl-1 (**a**, **b**, **c**, and **e**), Flag (**f**), PSMA5 (**g**), PSMD4/S5a (**g**), and poly ADP-ribose polymerase (PARP) (**h**) protein and Mcl-1 (**b**) mRNA were determined by western blotting and RT-PCR/qPCR, respectively. Actin served as a control for protein loadings. The values in panels (**b**, **d**, and **h**) represent the mean±S.D. from three independent samples. *P<0.05 compared with control. ^#^*P*<0.01 compared with ABT-737 plus cafestol. The data represent three independent experiments

To examine the apoptotic mechanisms facilitated by cafestol treatment, we checked whether cafestol could modulate Mcl-1 expression. As shown in Figures 4a and b, cafestol markedly decreased Mcl-1 expression within 3 h. In contrast, mRNA expression of Mcl-1 was not changed in cafestol-treated cells (Figure 4b, lower panel). Therefore, we subsequently investigated whether cafestol modulates stability of Mcl-1 protein. For this study, cells were treated with 20 *μ*g/ml cyclohexamide (CHX) in the presence or absence of cafestol for various time points. As shown in Figure 4c, cafestol inhibited the stability of the Mcl-1 protein. Because degradation of Mcl-1 is mainly modulated by proteasome,^24^ we checked whether proteasome-mediated Mcl-1 protein degradation occurs in cafestol-treated cells. First, the role of proteasome pathway in downregulation of Mcl-1 by cafestol was examined using the proteasome sensor vector, ZsProSensor-1. The proteasome sensor vector encodes a destabilized green fluorescence protein (ZsGreen), which is rapidly degraded by proteasomes. Cafestol reduced green fluorescence within 6 h (Figure 4d). This result indicated that cafestol induced proteasome activity. Next, we investigated whether proteasome inhibitors (MG132 and lactacystin) block cafestol-mediated downregulation of Mcl-1 expression. As shown in Figure 4e, both proteasome inhibitors markedly reversed cafestol-mediated inhibition of Mcl-1 expression. Next, to identify the role of ubiquitination in degradation of Mcl-1 protein, we used Flag-Mcl-1 and Flag-Mcl-1^KR^, in which all 14 lysine residues are replaced with arginine. As shown in Figure 4f, CHX and cafestol led to the rapid degradation of Mcl-1 proteins, and degradation of the Mcl-1^KR^ protein is slower than Mcl-1. Therefore these data indicated that ubiquitination is involved in degradation of Mcl-1, but ubiquitin-independent pathway might be also associated with degradation of Mcl-1 proteins. We further examined whether cafestol could modulate the expression of two important proteasome subunits, 20S proteasome subunit alpha type 5 (PSMA5) and 19S proteasome non-ATPase regulatory subunit 4 (PSMD4/S5a).^25^ However, cafestol had no effect on the expression of both proteins (Figure 4g). Finally, because downregulation of Mcl-1 have important role in cafestol plus ABT-737-mediated apoptosis, we investigated whether proteasome inhibitors could block apoptosis. Both proteasome inhibitors markedly inhibited cafestol plus ABT-737-induced sub-G1 population and PARP cleavage (Figure 4h). These results indicate that cafestol induces downregulation of Mcl-1 protein via activation of proteasome pathway.

### Upregulation of Bim expression is involved in cafestol-induced ABT-737-mediated apoptosis

**Figure 5:**
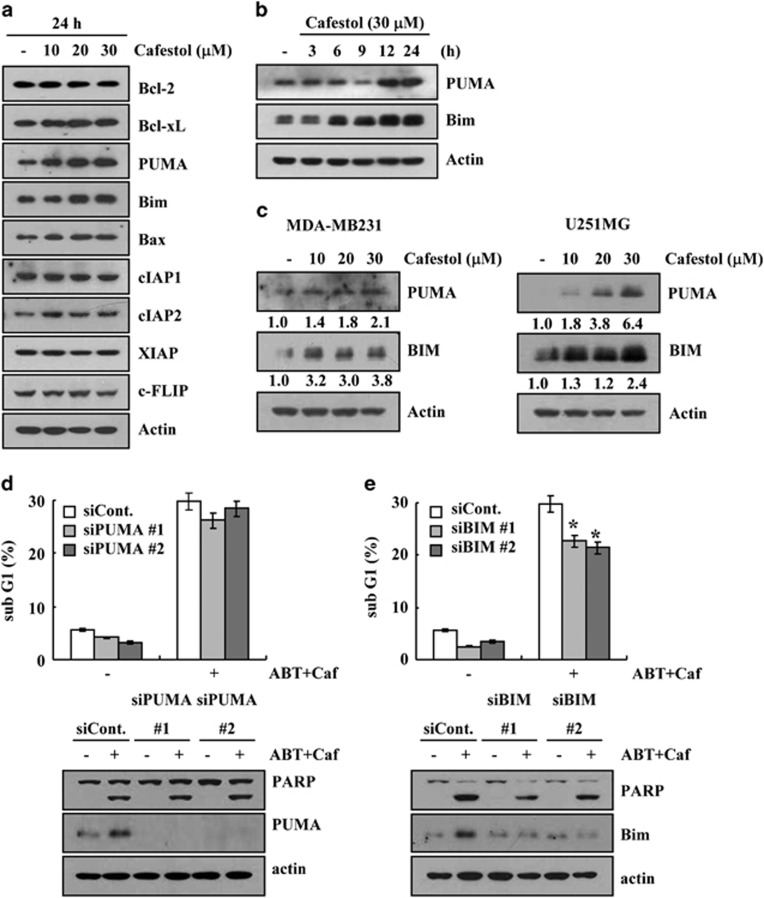
Upregulation of Bim expression is associated with cafestol plus ABT-737-induced apoptosis. (**a**) Caki cells were treated with the indicated concentrations of cafestol for 24 h. The protein expression levels of Bcl-2, Bcl-xL, PUMA, Bim, Bax, cIAP1, cIAP2, XIAP, and c-FLIP were determined by western blotting. Actin served as a control for protein loadings. (**b**) Caki cells were treated with 30 *μ*M cafestol for the indicated time periods. The protein expression levels of PUMA and Bim were determined by western blotting. Actin served as a control for protein loadings. (**c**) MDA-MB231 and U251MG cells were treated with the indicated concentrations of cafestol for 24 h. The protein expression levels of PUMA and Bim were determined by western blotting. Actin served as a control for protein loadings. (**d** and **e**) Mcl-1-overexpressed cells (Caki/Mcl-1) were transiently transfected with PUMA (**c**) and Bim (**d**) siRNA or control siRNA. Overnight after transfection, cells were treated with 30 *μ*M cafestol (Caf) and 0.1 *μ*M ABT-737 (ABT) for 24 h. The level of apoptosis was analyzed by measuring the sub-G1 fraction by flow cytometry (**d** and **e**, upper panel). Equal amounts of cell lysates (60 *μ*g) were separated by gel electrophoresis and analyzed by western blotting for poly ADP-ribose polymerase (PARP), PUMA, and Bim. Actin served as a control for protein loadings. The values in panels (**d** and **e**) represent the mean±S.D. from three independent samples. *P<0.05 compared with ABT-737 plus cafestol-treated control siRNA. The data represent three independent experiments

Next, we investigated whether cafestol regulated other apoptosis-related proteins. Although cafestol had no effect on other proteins, it dose-dependently increased the protein levels of PUMA and Bim (Figure 5a). Protein levels of PUMA were increased from 12 h of 30 *μ*M cafestrol treatment and those of Bim were enhanced from 6 h (Figure 5b). Furthermore, cafestol also dose-dependently increased the protein levels of PUMA and Bim in MDA-MB-231 and U251MG cells (Figure 5c). Therefore, we investigated whether these proteins were related to ABT-737 plus cafestol-induced apoptosis. Downregulation of PUMA expression by siRNA did not block apoptosis (Figure 5d). However, ABT-737 plus cafestol-induced apoptosis was markedly inhibited by downregulation of Bim expression by siRNA (Figure 5e). These data suggest that upregulation of Bim expression critically contributed to the sensitization of ABT-737-mediated apoptosis.

## Discussion

In this study, we show that cafestol acts as a potent enhancer of ABT-737-induced apoptosis in cancer cells, which have Mcl-1-mediated resistance to ABT-737. In addition, combined treatment with cafestol and ABT-737 markedly reduced tumor formation and induced apoptosis in a xenograft model. The main mechanism of cafestol-mediated ABT-737-induced apoptosis may be downregulation of Mcl-1 expression and upregulation of Bim expression.

ABT-737 binds to Bcl-xL, Bcl-2, and Bcl-w with high affinity (*K*_i_≤1 nM), while it binds to Mcl-1 with *K*_i_=1>*μ*M.^7^ Therefore ABT-737 could induce apoptosis in several cancer cells, which highly expressed anti-apoptotic Bcl-2 family. For example, ABT-737 markedly increased apoptosis in multiple myeloma,^26^ acute myeloid leukemia,^8^ chronic lymphocytic leukemia,^27^ and small cell lung cancer.^28^ However, resistance to ABT-737 in several types of cancer cell has been attributed to the high level of Mcl-1 expression. As ABT-737 does not bind Mcl-1 with low affinity, activity of Mcl-1 is not affected by ABT-737. Therefore many researchers have found drugs to overcome resistance to ABT-737-mediated apoptosis via downregulation of Mcl-1 expression. The synthetic cytotoxic retinoid *N*-(4-hydroxyphenyl) retinamide (4-HPR) decreased Mcl-1 expression via reactive oxygen species production, resulting in cytotoxicity in acute lymphoblastic leukemia.^29^ In addition, multiple drugs, such as quercetin,^30^ gemcitabine,^31^ actinomycin D,^32^ and histone deacetylase inhibitor entinostat,^33^ potentiates cancer cell to ABT-737-induced apoptosis by targeting Mcl-1. In our study, we found that cafestol also decreased Mcl-1 expression, resulting in increased sensitivity to ABT-737-mediated apoptosis.

In addition, cafestol markedly increased Bim and PUMA expression, pro-apoptotic BH3-only proteins (Figure 5b). BH3-only proteins, such as NOXA, PUMA, and Bim, bind to the anti-apoptotic Bcl-2 proteins, resulting in inhibition of apoptosis. Bim and PUMA could bind to Bcl-2, Bcl-xL, Bcl-w, and Mcl-1.^34^ Therefore, we thought that cafestol-induced Bim and PUMA expression was involved in cafestol plus ABT-737-mediated apoptosis. However, downregulation of Bim, but not downregulation of PUMA, reduced ABT-737 plus cafestol-induced apoptosis (Figures 5d and e). Upregulation of Bim expression was detected within 6 h, but PUMA was increased within 12 h. Therefore, upregulation of PUMA expression might be a result and not the cause. Our data suggested that cafestol could induce ABT-737-mediated apoptosis via two mechanisms; downregulation of Mcl-1 expression and upregulation of Bim expression.

Mcl-1 proteins can be regulated by transcriptional, translational, and posttranslational mechanisms. When protein synthesis was blocked by CHX, Mcl-1 expression dramatically decreased within 30 min, and cafestol facilitated Mcl-1 degradation (Figure 4c). Posttranslational regulation of Mcl-1 expression can be divided into the following three pathways. First, there is a rapid turnover of Mcl-1 via the ubiquitin/proteasome system.^24, 35^ Zhong *et al.*^24^ reported that Mcl-1 ubiquitin ligase E3 (MULE), as a E3 ubiquitin ligase, is responsible for the ubiquitination of Mcl-1. Another E3 ubiquitin ligase, which targets Mcl-1, is a beta transducin-containing protein (*β*-TrCP).^36^ Second, Mcl-1 is degraded by non-proteasomal pathway. Initially, Mcl-1 is cleaved by caspase-3 during apoptosis.^37, 38^ Caspase-3 induces cleavage of Mcl-1 at Asp-127 and Asp-157 in tumor necrosis factor-related apoptosis-inducing ligand-induced apoptosis of Jurkat T cells and hematopoietic cells. Then granzyme B also cleaves Mcl-1 in granzyme-mediated apoptosis of Jurkat cells.^39^ Third, lysosomal degradation pathway is involved in Mcl-1 degradation.^40^ FTY720 induces apoptosis of leukemic natural killer cells by degradation of Mcl-1 via lysosomal degradation pathway.^40^ In our study, proteasome inhibitors markedly reversed cafestol-mediated downregulation of Mcl-1 (Figure 4e) and ABT-737 plus cafestol-mediated apoptosis (Figure 4h). Furthermore, proteasome activity was increased in cafestol-treated cells (Figure 4d). Therefore, the induced proteasome activity by cafestol might increase the sensitivity to ABT-737-mediated apoptosis.

Collectively, these results suggest that cafestol sensitizes ABT-737-mediated apoptosis through the downregulation of Mcl-1 expression and upregulation of Bim expression in the human renal Caki cell lines. Therefore we suggest that cafestol may be effectively used as a sensitizer of ABT-737 in the treatment of solid tumors.

## Materials and Methods

### Cells and materials

Human renal carcinoma cells (Caki), human breast carcinoma cells (MDA-MB231), human glioma cells (U251MG), human colon carcinoma cells (HCT116), human leukemia cells (U937), and human prostate carcinoma (PC3) cells were obtained from the American Type Culture Collection (Manassas, VA, USA). Human ovarian carcinoma cells (A2780) was obtained from Sigma-Aldrich (St. Louis, MO, USA). HSF were a gift from Dr. TJ Lee (Yeungnam University, Gyeongsan, Korea). All cells were cultured in Dulbecco's Modified Eagle's Medium containing 10% fetal bovine serum, 20 mM HEPES buffer, 100 U/ml penicillin, 100 *μ*g/ml streptomycin, and 100 *μ*g/ml gentamicin. PCR primers were purchased from Macrogen (Seoul, Korea). ABT-737 was purchased from Sellek (Houston, TX, USA), and cafestol was purchased from LKT Laboratories (St. Paul, MN, USA). Resveratrol and curcumin were obtained from Biomol (Plymouth Meeting, PA, USA). z-VAD-fmk was obtained from Calbiochem (San Diego, CA, USA). Anti-DR5, anti-Bcl-2, anti-Bcl-xL anti-Mcl-1, anti-cIAP1, anti-cIAP2, and anti-PUMA antibodies were purchased from Santa Cruz Biotechnology (Santa Cruz, CA, USA). Anti-PARP antibody was purchased from Cell Signaling Technology (Beverly, MA, USA). Anti-XIAP and anti-Bax antibodies were purchased from BD Biosciences (San Jose, CA, USA). The anti-Bim antibody was purchased from Millipore Corporation (Billerica, MA, USA). Anti-c-FLIP(L) antibody was obtained from ALEXIS Corporation (San Diego, CA, USA). Anti-PSMD/S5a and anti-PSMA5 antibodies were purchased from Cell Signaling Technology. All other chemicals were purchased from Sigma-Aldrich.

### Stable transfection in Caki cells

The Caki cells were transfected in a stable manner with the pFLAG-CMV4-Mcl-1, pcDNA 3.1-Bcl-2 plasmid, or control plasmid pcDNA 3.1 vector using Lipofectamine2000 as prescribed by the manufacturer (Invitrogen, Carlsbad, CA, USA). After 48 h of incubation, transfected cells were selected in primary cell culture medium containing 700 *μ*g/ml G418 (Invitrogen). After 2 or 3 weeks, single independent clones were randomly isolated, and each individual clone was plated separately. After clonal expansion, cells from each independent clone were tested for expression levels of Mcl-1 and Bcl-2 by immunoblotting.

### Flow cytometry analysis

For flow cytometry, the cells were resuspended in 100 *μ*l of phosphate-buffered saline (PBS), and 200 *μ*l of 95% ethanol was added while the cells were being vortexed. Then the cells were incubated at 4 °C for 1 h, washed with PBS, resuspended in 250 *μ*l of 1.12% sodium citrate buffer (pH 8.4) with 12.5 *μ*g of RNase, and incubated for an additional 30 min at 37 °C. The cellular DNA was then stained by adding 250 *μ*l of a propidium iodide solution (50 *μ*g/ml) to the cells for 30 min at room temperature. Stained cells were analyzed by fluorescent-activated cell sorting on a FACScan flow cytometer to determine relative DNA content, which was based on red fluorescence intensity.

### Western blotting analysis

Cells were washed with cold PBS and lysed on ice in 50 *μ*l of lysis buffer (50 mM Tris-HCl, 1 mM EGTA, 1% Triton X-100, 1 mM phenylmethylsulfonyl fluoride, pH 7.5). Lysates were centrifuged at 10 000 × *g* for 10 min at 4 °C, and the supernatant fractions were collected. Proteins were separated by SDS-PAGE and transferred to an Immobilon-P membrane (Amersham, Uppsala, Sweden). Specific proteins were detected using an enhanced chemiluminescence (ECL) western blotting kit according to the manufacturer's instructions.

### Determination of synergy

The possible synergistic effect of cafestol and ABT-737 was evaluated using the isobologram method. In brief, cells were treated with different concentrations of cafestol and ABT-737 alone or in combination. After 48 h, relative survival was assessed, and the concentration effect curves were used to determine the IC_50_ (the half-maximal inhibitory concentration) values for each drug alone and in combination with a fixed concentration of the second agent.^41^

### 4′,6′-Diamidino-2-phenylindole staining (DAPI) for condensation and fragmentation of nuclei

To examine cellular nuclei, cells were fixed with 1% paraformaldehyde on glass slides for 30 min at room temperature. After fixation, cells were washed with PBS and a 300 nM DAPI solution (Roche, Mannheim, Germany) was added to the fixed cells for 5 min. After the nuclei were stained, cells were examined by fluorescence microscopy.

### Cell death assessment by DNA fragmentation assays

A cell death detection ELISA plus kit (Boehringer Mannheim, Indianapolis, IN, USA) was used for assessing apoptotic activity by detecting fragmented DNA within the nucleus in ABT-737-, cafestol-, and combination of ABT-737 and cafestol-treated cells. Briefly, each culture plate was centrifuged for 10 min at 200 × *g*, the supernatant was removed, and the pellet was lysed for 30 min. After centrifuging the plate again at 200 × *g* for 10 min, the supernatant that contained the cytoplasmic histone-associated DNA fragments was collected and incubated with an immobilized anti-histone antibody. The reaction products were incubated with a peroxidase substrate for 5 min and measured by spectrophotometry at 405 and 490 nm (reference wavelength) with a microplate reader (Tecan, Männedorf, Switzerland). The signals in the wells containing the substrate alone were subtracted as the background.

### Asp-Glu-Val-Asp-ase (DEVDase) activity assay

To evaluate DEVDase activity, cell lysates were prepared after their respective treatments with ABT-737 in the presence or absence of cafestol. Assays were performed in 96-well microtiter plates by incubating 20 *μ*g of cell lysates in 100 *μ*l of reaction buffer (1% NP-40, 20 mM Tris-HCl, pH 7.5, 137 mM NaCl, 10% glycerol) containing a caspase substrate (Asp-Glu-Val-Asp-chromophore-p-nitroanilide (DVAD-pNA)) at 5 *μ*M. Lysates were incubated at 37 °C for 2 h. Thereafter, the absorbance at 405 nm was measured with a spectrophotometer (Tecan).

### Clonogenic survival assay

Caki cells (0.5 × 10^5^) were seeded in a 12-well culture plates and was followed by treatment with cafestol and ABT-737 for 3 days. Clonogenic survival was determined by staining colonies using 0.4% coomassie blue and were visualized by a digital camera (Canon, Melville, NY, USA).

### Animal

Male BALB/c-nude mice, aged 5 weeks, were purchased from the Central Lab Animal Inc. (Seoul, Korea). All the mice were allowed 1 week to acclimatize to the surroundings before the experiments and were kept at 25±2 °C, with a relative humidity of 55±5% and a 12-h light–dark cycle. The study protocol was approved by the IRB Keimyung University Ethics Committee.

### *in vivo* xenograft model

Each mouse was s.c. injected on each flank with Mcl-1-overexpressing Caki cells (1 × 10^6^). After tumors had grown to at around 2 weeks, 28 mice were randomly divided into four treatment groups: (1) vehicle alone, (2) ABT-737 alone, (3) cafestol alone, and (4) ABT-737 plus cafestol. ABT-737 and cafestol were administered at 75 mg/kg, respectively. ABT-737 was prepared in 65% of 5% dextrose, 30% propylene glycol, and 5% Tween-80 (pH 3.5). Cafestol was prepared in corn oil. Mice received intraperitoneal (i.p.) injection of ABT-737 and cafestol. Treatment was administered twice a week for 2 weeks. Growth of the s.c. tumors was measured twice a week. Tumor size was measured twice a week using a Vernier's caliper (Mytutoyo Co., Tokyo, Japan) across its two perpendicular diameters, and tumor size was calculated using the equation (length × width^2^)/2. The animals were killed through cervical dislocation, and tumors were collected for histological analysis. The tumors were fixed in 30% formalin, embedded in OCT compound (Miles Inc., Elkhart, IN, USA), and cut into 20 *μ*m using cryostat (SLEE International, Inc., New York, NY, USA).

### TUNEL assay

Apoptosis in tumor cells was detected by TUNEL assay. It was performed using the ApopTag Fluorescein *In Situ* Apoptosis Detection Kit (Millipore) as per the manufacturer's protocol.

### Immunohistochemistry

Sections were mounted on gelatin-coated slides, dried for 1 h, and washed twice in PBS. This was followed by blocking with 1% BSA and incubation with active caspase-3 antibody (Cell Signaling Technology). DAPI (Vector Laboratories, Burlingame, CA, USA) was used to detect nuclei. Sections were photographed using a Carl Zeiss microscope (Carl Zeiss, Jena, Germany).

### Reverse transcription-PCR

Total RNA was isolated using the TriZol reagent (Life Technologies, Gaithersburg, MD, USA), and cDNA was prepared using M-MLV reverse transcriptase (Gibco-BRL, Gaithersburg, MD, USA) according to the manufacturer's instructions. The following primers were used for the amplification of human Mcl-1 and actin: Mcl-1 (forward) 5′-GCGACTGGCAAAGCTTGGCCTCAA-3′ and (reverse) 5′-GTTACAGCTTGGATCCCAACTGCA-3′ and actin (forward) 5′-GGCATCGTCACCAACTGGGAC-3′ and (reverse) 5′-CGATTTCCCGCTCGGCCGTGG-3′. PCR amplification was carried out using the following cycling conditions: 94 °C for 3 min followed by 17 cycles for actin, or 25 cycles for Mcl-1 of 94 °C for 40 s, 58 °C for 40 s, 72 °C for 40 s, and a final extension at 72 °C for 5 min. The amplified products were separated by electrophoresis on a 1.5% agarose gel and detected under UV light by the addition of ethidium bromide. For qPCR, cDNA and forward/reverse primers (200 nM) were added to 2 × KAPA SYBR Fast master mix, and reactions were performed on RG-6000 real-time amplification instrument (Corbett Research, Cambridge, UK). The following primers were used for the amplification of human Mcl-1 and actin: Mcl-1 (sense) 5′-ATGCTTCGGAAACTGGACAT-3′ and (antisense) 5′-TCCTGATGCCACCTTCTAGG-3′, and actin (sense) 5′-CTACAATGAGCTGCGTGTG-3′ and (anti-sense) 5′-TGGGGTGTTGAAGGTCTC-3′. Threshold cycle number (Ct) of each gene was calculated, and actin was used as reference genes. Delta-delta Ct values of genes were presented as relative fold induction.

### Proteasome activity assay

Measurement of proteasome activity within cells was determined using ZsProSensor-1 (proteasome sensor vector) (BD Biosciences). The ZsProSensor-1 stable cell line was created by transfection of Caki cells with the ZsProsensor-1 plasmid using Lipofectamine (Invitrogen). Clones were selected in the presence of 700 *μ*g/ml G418 (Gibco-BRL), and fluorescence was detected using FACS Canto (BD Biosciences).

### Small-interfering RNAs (siRNAs)

The GFP (control), PUMA, and Bim siRNA duplexes used in this study were purchased from Santa Cruz Biotechnology and Dharmacon (Lafayette, CO, USA). Cells were transfected with siRNA oligonucleotides using Oligofectamine Reagent (Invitrogen) according to the manufacturer's recommendations.

### Plasmids

Flag-Mcl-1 (plasmid number: 32978) and Flag-Mcl-1^KR^ (plasmid number: 32979), which was deposited by Stewart, was purchased from Addgene (Cambridge, MA, USA).^42^

### Statistical analysis

The data were analyzed by using a one-way ANOVA and *post-hoc* comparisons (Student–Newman–Keuls) using the Statistical Package for Social Sciences 22.0 software (SPSS Inc., Chicago, IL, USA).

## Abbreviations

CHX: cyclohexamide
PSMA5: 20S proteasome subunit alpha type 5
PSMD4/S5a: 19S proteasome non-ATPase regulatory subunit 4
PUMA: p53 upregulated modulator of apoptosis
Bim: B-cell lymphoma 2 interacting mediator of cell death
