# Supplementary figures and images for: Cafestol overcomes ABT-737 resistance in Mcl-1-overexpressed renal carcinoma Caki cells through downregulation of Mcl-1 expression and upregulation of Bim expression
Source: Cell Death Dis. 2014 Nov 6;5(11):e1514–. doi: 10.1038/cddis.2014.472 (PMC4260730; doi:10.1038/cddis.2014.472)

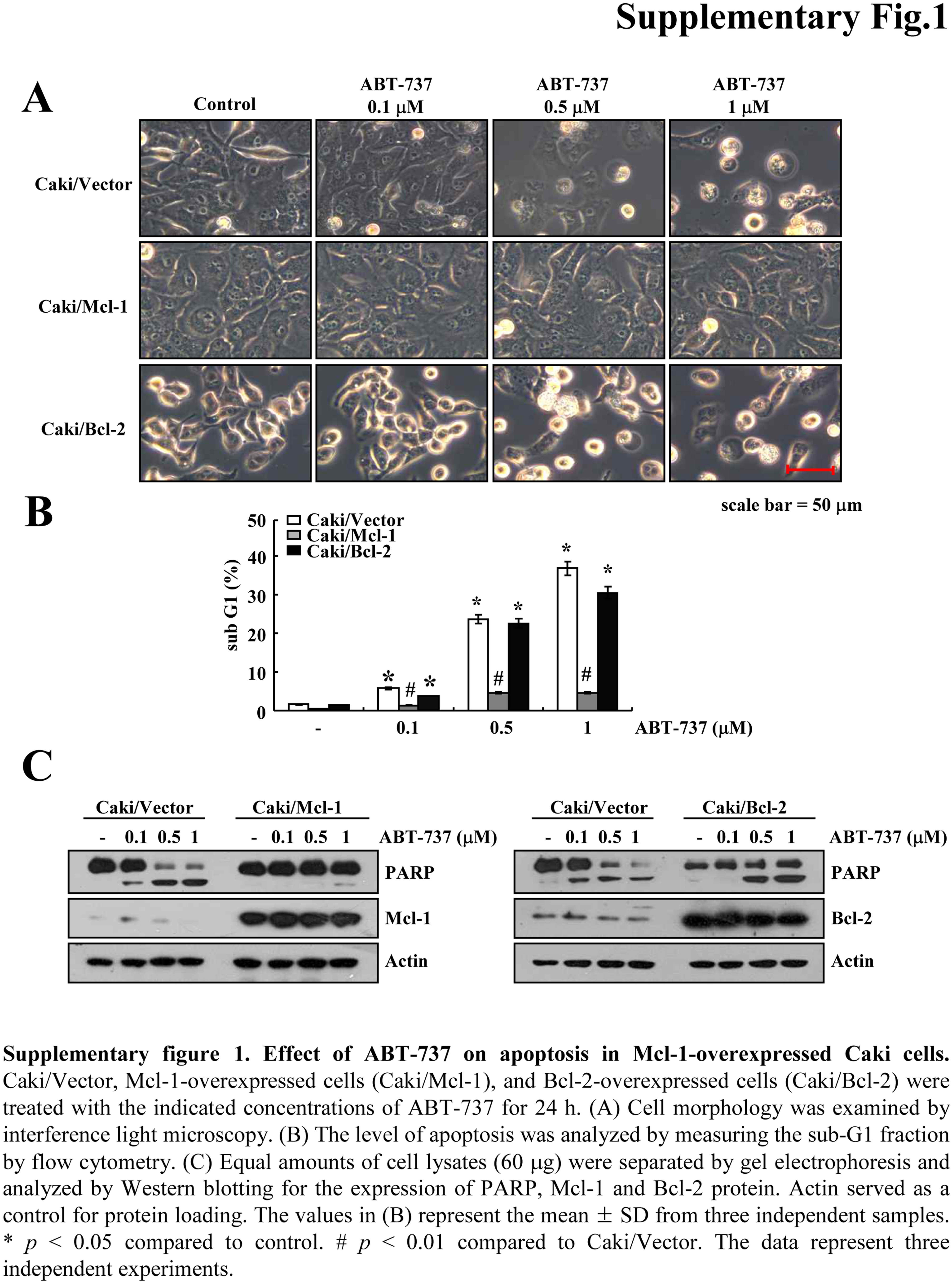

Supplement: Supplementary Figure 1 [file cddis2014472x1.tif]
